# Metabolic Biomarkers of Squamous Cell Carcinoma of the Aerodigestive Tract: A Systematic Review and Quality Assessment

**DOI:** 10.1155/2020/2930347

**Published:** 2020-02-21

**Authors:** Yan Mei Goh, Stefan S. Antonowicz, Piers Boshier, George B. Hanna

**Affiliations:** Department of Surgery & Cancer, Imperial College London, London W2 1NY, UK

## Abstract

*Introduction.* Aerodigestive squamous cell carcinomas (ASCC) constitute a major source of global cancer deaths. Patients typically present with advanced, incurable disease, so new means of detecting early disease are a research priority. Metabolite quantitation is amenable to point-of-care analysis and can be performed in ASCC surrogates such as breath and saliva. The purpose of this systematic review is to summarise progress of ASCC metabolomic studies, with an emphasis on the critical appraisal of methodological quality and reporting.

**Method:**

A systematic online literature search was performed to identify studies reporting metabolic biomarkers of ASCC. This review was conducted in accordance with the recommendations of the Cochrane Library and MOOSE guidelines.

**Results:**

Thirty studies comprising 2117 patients were included in the review. All publications represented phase-I biomarker discovery studies, and none validated their findings in an independent cohort. There was heterogeneity in study design and methodological and reporting quality. Sensitivities and specificities were higher in oesophageal and head and neck squamous cell carcinomas compared to those in lung squamous cell carcinoma. The metabolic phenotypes of these cancers were similar, as was the kinetics of metabolite groups when comparing blood, tissue, and breath/saliva concentrations. Deregulation of amino acid metabolism was the most frequently reported theme.

**Conclusion:**

Metabolite analysis has shown promising diagnostic performance, especially for oesophageal and head and neck ASCC subtypes, which are phenotypically similar. However, shortcomings in study design have led to inconsistencies between studies. To support future studies and ultimately clinical adoption, these limitations are discussed.

## 1. Introduction

Squamous cell carcinomas of the aerodigestive tract (ASCC) constitute a major health burden globally, with an estimated 4.3 million new cases and 2.6 million deaths annually [1]. Poor survival that is associated with ASCC reflects their often delayed presentation to medical professionals, such that many patients are not suitable for curative therapy [2–5]. Whilst the ability to diagnose ASCC at an early stage is associated with improved long-term survival, current strategies have inadequate diagnostic performance and are not recommended in national guidelines. There remains an unmet clinical need to develop reliable noninvasive and cost-effective methods for the early detection of ASCC.

ASCC arise from nonkeratinising stratified squamous epithelium lining the upper digestive tract (lips to lower oesophagus) and respiratory tract. This convenient location renders ASCC suitable for noninvasive testing using breath and saliva. The use of proteomics and genomics has historically been at the forefront of diagnostic studies. However, these techniques provide monothematic information and are less suited to point-of-care technologies needed for large-scale application [6]. Metabolites may be more appealing as they are amenable to noninvasive sampling and translatable to point-of-care analytical tools [7]. For example, in upper gastrointestinal adenocarcinoma (the other major ASCC subtype), exhaled metabolites have demonstrated promise for detecting treatable disease stages [8–10]. However, progress in this field has been hampered by inadequate standardisation, inconsistent quality assurance, and evolving analytical technology [11–14].

The purpose of this systematic review is to summarise progress of ASCC metabolomic studies. The specific objectives are (i) to assess methodological quality, (ii) to summarise the discriminatory performance of the proposed metabolic biomarkers, and (iii) to describe emerging metabolic themes for these cancers.

## 2. Materials and Methods

### 2.1. Literature Search

This review set out to identify all studies that measured differences in metabolites between patients with ASCC and relevant controls. A systematic literature search was conducted in accordance with the recommendations of the Cochrane Library and MOOSE guidelines [15]. The following databases were searched: Medline (1946–present) via OvidSP, Ovid Embase (1947–18^th^ January 2019), and Cochrane Library. Three strings using the following search terms were used: biomarkers; metabonomics; metabolic profiling; volatile organic compounds; magnetic resonance spectroscopy; mass spectrometry; and squamous cell carcinoma. All variations in spelling including a truncated search term using wild card characters and “related articles” function were used in combination with the Boolean operators AND OR. Full details of the search strategy were provided as a supplementary file. The reference lists of identified articles were also searched to identify other potentially relevant studies.

Two independent reviewers (YMG, PB) screened the titles and abstracts of all studies identified by the primary electronic search. The full texts of potentially relevant articles were retrieved to assess eligibility for inclusion. Included studies were those where metabolomic techniques to identify biomarkers of ASCC were performed in treatment-naïve human subjects. Studies were excluded if they reported on mixed cancer subtypes where results for ASCC could not be separately determined. Studies that did not report named biomarkers of ASCC, animal and in vitro studies, studies not published in the English language, and review articles and conference abstracts were also excluded. A third reviewer (SA) was consulted in the case of a disagreement.

### 2.2. Definitions

Metabolomics is defined as “the global and unbiased definition of the complement of small molecules in biofluids, tissues, organs, or organisms” [16]. Biomarkers were defined as a naturally occurring molecule, which were significantly different in a disease state. ASCC included tumours affecting squamous mucosa of the oral cavity, oropharynx, lung, and oesophagus.

### 2.3. Outcome Measures

The following data items were extracted from included publications: year of publication, country of origin, study design, recruitment time, total number of participants, tumour of origin, biomarker phase, tumour stage, analytical platform used, sample type, number of compounds identified, compounds noted to be increased/decreased in cancer, statistical analysis performed, prediction model used, sensitivity and specificity, and area under the receiver operating characteristic (ROC) curve derived from diagnostic models.

### 2.4. Statistical Analysis

Statistical analysis was performed using R (version 3.2.1, The R Project for Statistical Computing, http://www.r-project.org). Using the sensitivity, specificity, and area under the ROC curves derived from individual published models, bivariate meta-analyses were performed to create pooled point estimates of the hierarchal summary ROC curve of VOC analysis in accordance with previously validated methods [17].

### 2.5. Metabolite Analysis

**Figure 1:**
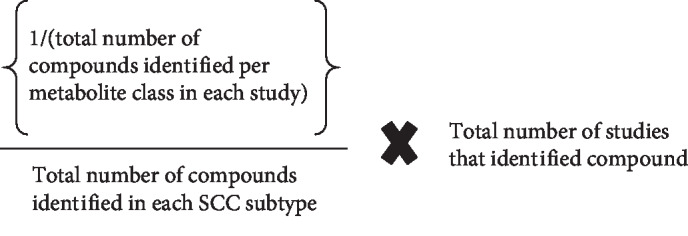
Equation for weighted means of each identified metabolite. Key: SCC: squamous cell carcinoma.

All metabolites identified were classed in accordance to the Kyoto Encyclopedia of Genes and Genomes (KEGG) pathway, and statistical analysis was performed using the pathway analysis module in MetaboAnalyst version 4.0. Metabolites determined to be significantly increased or decreased in each study were selected. Data preprocessing included name check against the Human Metabolome Database (HMDB), data checks, and missing values. Parameters used to analyse this data were the hypergeometric test for overrepresentation analysis and the relative-betweenness centrality test for pathway topology analysis based on the KEGG pathway library [18–20]. Normalisation was performed using the weighted means of identified metabolite that were increased/decreased in squamous cell carcinoma (SCC) in each sample type. The mean proportion of each compound identified was analysed as the proportion of the total number of compounds identified per metabolite class per study, divided by the total number of compounds identified in total in each SCC site subtype, multiplied by the total number of studies; this compound was identified in Figure 1.

### 2.6. Quality Assessment

Study quality was assessed with three tools: first, Quality Assessment of Diagnostic Accuracy Studies-2 (QUADAS-2) checklist [21] to assess methodological bias. Second, the Standards for Reporting of Diagnostic Accuracy Studies (STARD) checklist [22, 23] was used to assess general reporting quality of a clinical diagnostic tool. Third, the Chemical Analysis Working Group- (CAWG-) Metabolomics Standard Initiative (MSI) criteria was used as this focused on the reporting quality of metadata of metabolomic studies [14]. The CAWG-MSI Metabolite Identification Levels were used to summarise studies' identification rigour: level 1 (most confident, at least two orthogonal analytical data types, e.g., retention time, isotope labelling), level 2 (one data type, spectral similarity to commercial library), level 3 (one data type related to a spectral or chemical property).

## 3. Results

**Figure 2:**
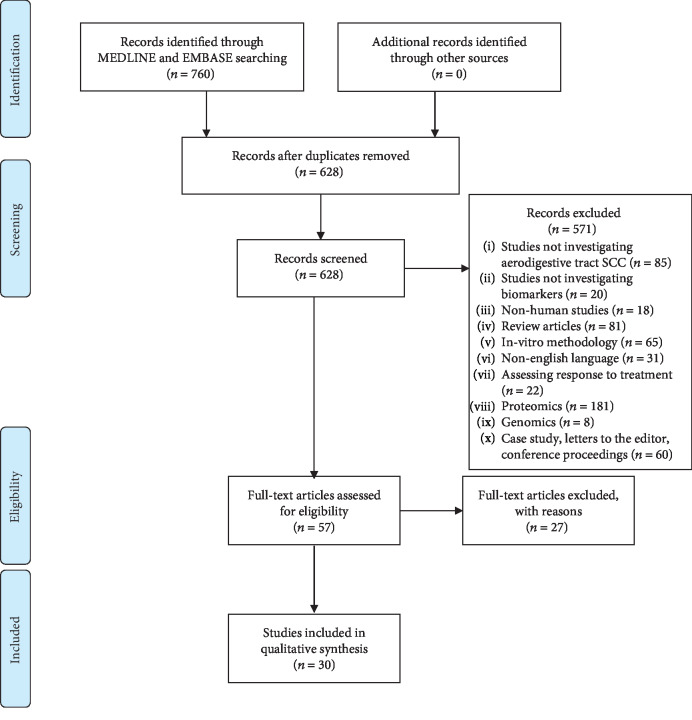
PRISMA chart.

A systematic literature search identified a total of 30 studies comprising of a total of 2117 subjects of which 1144 had a diagnosis of ASCC (Figure 2). Details of included studies were provided in Table 1. All studies were Phase I biomarker discovery studies. Of the 30 included studies, 18 were from Asia and the Far East [8, 9, 11–26], seven from Europe [27–33], three from North America [34–36], and two from the Middle East [37, 38]. ASCC tumour sites identified were the head and neck (*n* = 17), oesophageal (*n* = 8), and lung (*n* = 5). The majority of studies compared patients with cancer to normal controls and or benign conditions [16, 25, 26, 28, 29, 34, 38–43].

Liquid chromatography mass spectrometry (LC-MS) was the most commonly used analytical platform (*n* = 14) followed by gas chromatography mass spectrometry (GC-MS, *n* = 12). Sample types used in these studies were tissue (*n* = 10), saliva (*n* = 13), plasma (*n* = 13), urine (*n* = 5), and breath (*n* = 6). Several studies used more than one analytical platform [34, 35, 38, 40] and/or sample types for analysis [34, 35] (Table 1). Eight studies used targeted methods, and 22 studies untargeted methods. All studies that used untargeted methods covered a large range of commonly identified metabolites, ranging from small fatty acids to larger glycolipid and carbohydrate metabolites. Only five studies identified volatile compounds [26, 30, 36–38].

### 3.1. Quality

Assessment of bias and applicability of outcomes were analysed with QUADAS-2 (Table 1). The QUADAS-2 was divided into risk of bias of the following: patient selection, diagnostic test, reference standard, and patient flow and timing. Additionally, this test investigated applicability of patient selection, diagnostic test, and reference standard to the systematic review. There was an overall low risk of bias of these diagnostic tests and high applicability of these studies to the review question. In this QUADAS-2 analysis, the nature of patient flow and timing of sample analysis was least reported in studies in this review (*n* = 11) [16, 28–30, 33, 36, 39, 40, 44].

General reporting quality of a clinical diagnostic tool was assessed by the STARD checklist (Table 1). The STARD score for reported studies ranged from 21 to 37 with a mean of 29.4 (±4.76 S.D) where the maximum score is 41. More than 75% of studies reported inclusion and exclusion criteria, described the reference test and standards, and reported potential bias and analysis of diagnostic accuracy well. However, more than two thirds of studies failed to clearly demonstrate patient recruitment protocol, specifically, how patients were identified and recruited, the nature of recruitment, e.g., consecutive or random series and [22, 37, 40, 41, 45–47] sample size estimation [35, 42, 48], participant flow [24, 31, 35, 39, 49, 50], and adverse effects as a consequence of the diagnostic tool.

Reporting of clinical demographics was not consistent in each study. Of the 30 studies, only 10 fully reported all clinical demographics [28, 30, 34, 35, 38, 41, 43, 47, 49, 51], 13 reported at least patient age, gender, and clinical stage [16, 25, 26, 31, 36, 39, 40, 42, 45, 46, 48, 52, 53]. Seven studies did not report differences in metabolite profile at different tumour stages [24, 27, 29, 32, 36, 37, 46]. In these seven studies, four compared differences in metabolic profile between cancer and noncancer cohorts [24, 27, 32, 37].

Definitions of normal control differed most in tissue sample analysis, where Zhang et al. specified normal adjacent control tissue samples a minimum of 5 cm from the tumour site [54] in contrast to the other five tissue studies that used adjacent normal controls [26, 29, 45, 46, 53] without demonstrating adequacy of tissue clearance. Of all 30 studies in this review, only Shoffel-Havakuk et al. used patients with benign histology as controls [37]. No tissue study used normal samples from patients with no endoluminal pathology, which is pertinent as metabolic field effects exist in endolumens [55]. Various exclusion criteria were given to control donors' characteristics, including use of nonsteroidal anti-inflammatory drugs within the past week, antibiotic treatment and consumption of specific food, history of mucosal disorder, chronic and/or systemic disease such as diabetes, autoimmune disorders, heart disease, infection, and liver disease. Twenty-six studies involving biofluids or breath used healthy volunteer controls, one additionally used patients with benign diseases [24, 25, 27, 28, 30–43, 46–49, 51, 52, 56, 57]. The definition for healthy volunteers was based on history (six studies) or endoluminal study (18 studies).

Reporting of metadata in metabolomics datasets was assessed using CAWG-MSI [14] (Supplementary [Supplementary-material supplementary-material-1]). A summary of the minimum reported metadata is summarised in Table 1. Twenty of the 30 studies included in this systematic review used relative quantification of compounds [16, 24, 25, 28, 29, 31, 33–38, 40–43, 45, 48, 49, 52], whilst 10 included studies provided absolute quantification of compounds [27, 30, 32, 39, 46, 47, 51–53, 56]. Despite the availability of reporting guidelines for metabolomics analysis, only three studies reported greater than 50% of the CAWG-MSI criteria [34, 39, 52]. Overall, studies reported sample preparation, experimental analysis, and instrumental performance well. However, the majority (80%) did not provide method validation data [16, 24, 25, 27, 28, 30–38, 40–42, 45–49, 51, 52, 54, 56]. Thirteen studies that analysed relative quantification of metabolites identified used either internal standards or normalised the results to allow for instrument variation [16, 24, 25, 29, 33–36, 40–42, 45, 49]. Six of the 10 studies that used absolute quantification did not report accuracy or precision validation data for their method on the instrument [30, 44, 46, 47, 51, 56] whilst two of 10 studies reported the limits of quantification and detection of their method [39, 52]. Out of 30 studies, only 12 declared evidence of data preprocessing [25, 29, 33, 34, 36, 37, 40, 41, 43, 45, 48, 49]. Levels one, two, and three metabolite identification were reported in nine [25, 27, 30, 32, 36, 39, 40, 46, 52], 15 [16, 24, 26, 29, 33, 34, 37, 42, 43, 45, 47, 49, 51, 56, 58], and six [28, 31, 35, 38, 41, 48] studies, respectively. Only two of the 30 studies reported all of the statistical aspects suggested by the CAWG-MSI guidelines [59].

### 3.2. Discriminatory Features

The highest sensitivity of oesophageal squamous cell cancer (OSCC) diagnosis was reported by Zhang et al. at 97.4% with a specificity of 95% and AUC of 0.988 [44]. Jin et al. reported the highest specificity at 96.67% with a sensitivity of 90% and AUC of 0.964 [42]. The highest sensitivities and specificities of lung squamous cell cancer (LSCC) were poorer with Handa et al. reporting the highest sensitivity of 97.4% [28] and Sanchez-Rodriguez et al. reporting the highest specificity of 68% and AUC of 0.7 [30]. The highest sensitivity, specificity, and AUC were reported for head and neck squamous cell cancer (HNSCC): 100%, 96.7%, and 0.997, respectively (Table 1). However, no groups subsequently validated their initial findings in independent cohorts. Of the 6 studies which reported AUC > 0.90, a high risk of bias was not present and CAWG-MSI metabolite identification was level 1 or 2.

### 3.3. Metabolic Themes

A total of 181 metabolites identified were associated with an increase or decrease in concentration in patients with ASCC compared to their normal controls (Supplementary [Supplementary-material supplementary-material-1]). These compounds were identified in a range of sample types including tissue, plasma, urine, saliva, and breath. The majority were amino acids, carboxylic acids, or fatty acids, and these were more commonly identified in tissue, saliva, and plasma samples. The least common metabolites identified were vitamins, nitrogen, and sulphur containing compounds (Supplementary [Supplementary-material supplementary-material-1]). Sixty-eight compounds that changed in ASCC were reported in more than one study. These metabolites were selected based on metabolites that were identified to be increased or decreased in cancer in different studies. Of these, 27 compounds were noted to be involved in amino acid and lipid metabolism (Supplementary [Supplementary-material supplementary-material-1]). All biomarkers showed a consistent increase or decrease in the sample types across different studies (see Supplementary Tables [Supplementary-material supplementary-material-1] and [Supplementary-material supplementary-material-1]).

**Figure 3:**
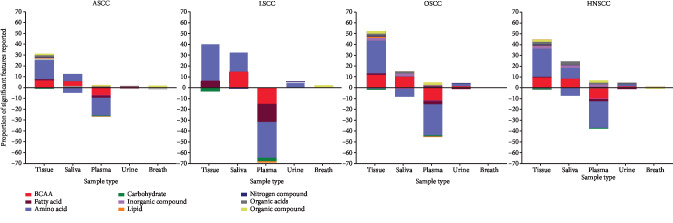
Proportion of identified compounds in each ASCC, LSCC, OSCC, and HNSCC in different sample types. Key: ASCC: aerodigestive squamous cell carcinoma; OSCC: oesophageal squamous cell carcinoma; LSCC: lung squamous cell carcinoma; HNSCC: head and neck squamous cell carcinoma; BCAA: branched chain amino acid.

**Figure 4:**
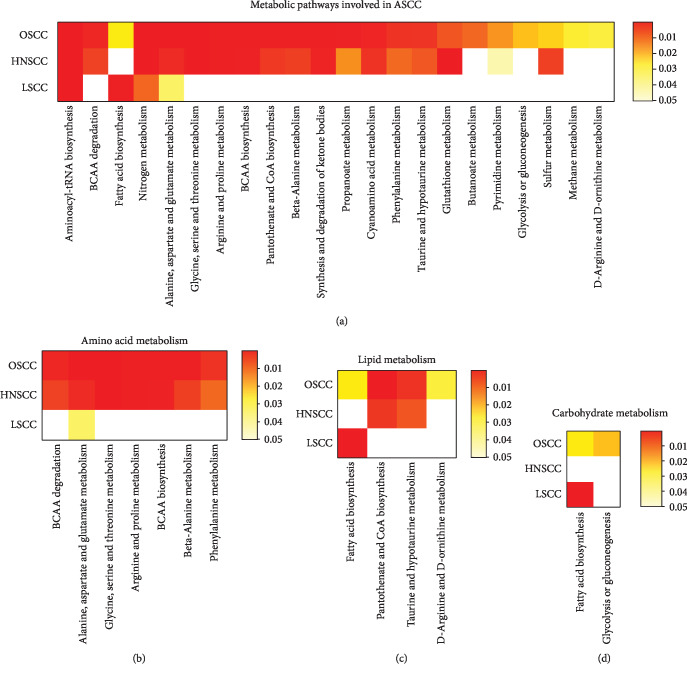
Metabolic pathways involved in all ASCC: (a) all metabolic pathways, (b) amino acid metabolism, (c) lipid metabolism, and (d) carbohydrate metabolism.

A particularly deregulated pathway was branched chain amino acid metabolism (BCAAs, see Figures 3 and 4 and Supplementary [Supplementary-material supplementary-material-1]). There were 36 significant differences in BCAA concentrations, or their downstream metabolites, across 12 studies [25, 28, 34–36, 39–41, 43, 45, 46, 49]. The QUADAS-2 risk of bias was low for nine of these studies [34–36, 40, 41, 45–47, 49]. Five of these studies were of good quality and five of fair quality as assessed by the STARD checklist. One study reported the minimum metadata required from the CAWG-MSI checklist [39]. Of these 12 studies, two reported level 1 metabolite identification [35, 39], seven reported level 2 [25, 28, 36, 41, 43, 45, 60], and three reported level 3 metabolite identification [34, 40, 46].

### 3.4. Influence of Anatomical Location on Metabolic Themes

LSCC (*n* = 9) had the lowest number of metabolite classes compared to OSCC (*n* = 20) or HNSCC (*n* = 18) (Figure 3). Common metabolites that were identified in all ASCC sites were amino acids, fatty acids, carbohydrate, nitrogen compounds, and organic acids. OSCC and HNSCC appear to demonstrate similar metabolic profiles compared to LSCC (see Figures 3 and 4). Metabolic pathways commonly deregulated in both OSCC and HNSCC mainly concerned amino acid mobilisation, uptake, and polymerisation; lipid synthesis; and alternative energy. All twelve studies demonstrating BCAA deregulation were either OSCC or HNSCC. These compounds were increased in tissue and saliva but decreased in cancer patients' plasma. Breakdown products of BCAA, alpha-ketoisocaproic acid (KIC), alpha-ketoisovaleric acid (KIV), and alpha-ketomethylvaleric acid (KMV) were reported in three metabolomic studies [35, 42, 48] related to OSCC and HNSCC. However, the design and reporting heterogeneity meant these site-specific results should be approached with caution, and further detailed analyses were not performed.

### 3.5. Influence of Biosample Type on Metabolic Themes

There was an overall positive deflection in the proportion of metabolites present in the tissue, saliva, urine, and breath of ASCC patients and a negative deflection in plasma (Figure 3). This was particularly evident for amino acids. Metabolites in ASCC saliva samples were more similar to tissue than plasma. In particular, increased BCAAs were identified in tissue and saliva of patients with ASCC. In contrast, plasma BCAAs were decreased in ASCC plasma and not identified in urine or breath. This trend was also noted in other amino acids. BCKAs were decreased in the plasma of ASCC patients, but the proportions of fatty acids increase and decrease were similar in tissue samples. However, the design and reporting heterogeneity meant these sample-specific results should be approached with caution, and further detailed analyses were not performed.

## 4. Discussion

This systematic review provides an overview of progress in ASCC metabolic biomarker studies. The principal findings of this review were (i) favourable diagnostic performance of metabolic biomarkers for the detection of OSCC and HNSCC but not LSCC in pooled analysis, (ii) shared metabolic features of OSCC and HNSCC, and (iii) suggestion of a consistent role of the KEGG amino acid metabolic pathway in ASCC. Additionally, comparing sample types suggests metabolites are often depleted in the circulation and enriched in both tumour tissue and luminal surrogates, suggesting a model for ASCC biomarker kinetics. From the design perspective, clinical methodology and reporting quality was of a reasonable standard, but analytical methodology and reporting quality were often of a poor standard, and no studies performed exceptionally in both aspects.

ASCCs all have high mortality due to late disease detection. Currently, there are no screening strategies for any subtype of sufficient accuracy and quality to support political endorsement. Pooled analysis of identified studies regarding the detection of ASCC gave an area under the curve (AUC) of 0.927 with sensitivity of 85.7% (95% CI 78.9–92%), respectively. This diagnostic performance compares favourably to existing screening programmes such as faecal occult blood testing for colorectal cancer and cytological cervical screening test that currently are associated with lower sensitivity and specificity [61, 62]. Although the studies were generally of an exploratory nature without extensive validation, these results are encouraging and suggest metabolic biomarkers of ASCC may provide novel screening tools to identify high-risk populations, provided these efforts can progress to high-quality validation studies with appropriate power. The finding that the six studies reporting the highest AUC values had good clinical design and used targeted metabolomic methods suggests methodological rigour and hypothesis-driven metabolomics generate the best results.

In both discriminatory performance and metabolic themes, HNSCC and OSCC clustered away from LSCC, suggesting the underlying biology of those cancers is better suited to metabolic biomarker studies. Both HNSCC and OSCC arise from normally resident squamous cells, whereas LSCC arise from metaplastic squamous cells, perhaps explaining LSCCs' relative metabolic heterogeneity. Moreover, genomic studies suggest LSCC to be distinct from HNSCC and OSCC [63–65]. Nonetheless, the relatively lower number of quantified metabolites for LSCC suggests this cancer warrants further study, perhaps using the similar analytical approaches from the best OSCC/HNSCC studies.

Despite using weighting to account for multiplicity from untargeted approaches, the heterogeneity in study design and quality, and the lack of independent validation, made comprehensive biological interpretation of the observed metabolic difference speculative. An exception to this was BCAA metabolism, which was a consistent theme in across the ASCC subtypes. There were 36 significant differences in BCAA concentrations or their downstream metabolites, across 12 studies [25, 26, 28, 34–36, 41–43, 46, 56, 66]. This was far more than any other metabolite group. Increased BCAAs were observed in ASCC tissue samples from four studies [26, 36, 45, 53], and decreased BCAA levels in cancer-blood samples were observed in two studies [42, 56]. These differences were often stark. This suggests uptake of BCAAs into ASCCs against the concentration gradient. BCAAs constitute 35-40% of human protein and are thus essential amino acids necessary for protein synthesis in rapidly dividing cells [67]. They also have additional proproliferative effects. For example, leucine potently activates the mammalian target of rapamycin complex 1 [60, 68–70] and BCAA deamination is a major source of glutamine for alternative energy [68, 71]. Thus, BCAA metabolism is emerging as critical mediators of transformation and treatment escape in a number of malignancies including other squamous cancers [72, 73] and the present finding of consistent BCAA reprogramming in ASCC warrants further targeted study.

Metabolomic biomarker analytics has evolved considerably in the last 15 years, and that progress is reflected in the design heterogeneity of the included studies. Critical appraisal of analytical design using CAWG-MSI generally revealed a low standard. In contrast, only six of the studies included in this review demonstrated poor STARD/QUADAS-2 scores (score of less than 25), indicating a reasonable quality of clinical design and reporting (Supplementary [Supplementary-material supplementary-material-1]). A key issue with metabolomic studies is the compromise between metabolomic coverage and unambiguous compound identification. Several studies used untargeted methodologies [16, 25, 26, 28, 29, 31, 33–38, 40–43, 45, 47–49, 51, 53] or more than one platform [3, 8, 9, 12] to increase their metabolomic coverage, although none used ultra-high coverage techniques such as Fourier-transform ion cyclotron resonance mass spectrometry (FT-ICR). No studies did not meet their objectives, or overstated their conclusions; however, this suggests that significant aspects of the ASCC metabolome have not been explored. Six studies that performed targeted methodology achieved level 1 identification of compounds of interest [27, 30, 32, 39, 46, 52], and two further studies used only commercially available spectral libraries for confirmation of their compound of interest [24, 56].

Additionally, our critical review has highlighted the following recurrent shortcomings in the current ASCC metabolomic literature: (i) lack of a clear sample size calculation; (ii) poor description of patient recruitment and inadequate description of clinical metadata; (iii) poor description of method validation; (iv) inconsistent quality assurance, especially replicate analysis; (v) biomarker performance frequently reported as multivariable models rather than clinical metrics; and (vi) lack of model validation data, either using internal cross-validation, or independent validation cohorts or studies. Using the CAWG-MSI checklist during study design would help to mitigate these issues [14].

A potential limitation was that more patients included in this review had late-stage disease (*n* = 548) rather than early-stage disease (*n* = 331), and that the case mix was usually just reported rather than subject to subgroup analysis. Typically, the clinical motivation for the work was early cancer detection, which seems at odds with test populations enriched for late-stage disease, without subgroup analysis. However, the majority of these studies were performed in tertiary centre settings, which meant that patients would typically have been on a curative pathway. Thus, the observed metabolic differences can detect treatable disease, which provides a platform for further studies powered to detect truly early disease. It should also be noted that more than half of the articles in this review were performed in China and Japan and may not be applicable to Western populations.

## 5. Conclusion

This review summarised progress in using metabolites to identify patients with ASCC. There was significant heterogeneity in methodology and quality; however, especially for OSCC and HNSCC, metabolites showed promise for minimally invasive diagnosis. These two ASCC subtypes had similar metabolic phenotypes, with deregulation of amino acid metabolism particularly pronounced. Comparative analysis of different sample types suggested a kinetics model for amino acids across the endolumen. To aid the development of future studies and ultimately clinical translation, the summarised recurrent methodological weaknesses must be addressed, especially with respect to analytical design.

## Figures and Tables

**Table 1:** Study characteristics, statistical analysis, and prediction model performed.

Author	Country	Sample type	SCC stage	Targeted/untargeted method	Analytical platform	Statistical analysis/prediction model	STARD score	QUADAS		CAWG-MSI metabolite ID level	CAWG-MSI score	Sn (%)	Sp (%)	AUC
								Risk of bias	Applicability					
Studies of oesophageal squamous cell carcinoma														
Liu 2013	China	Plasma	Late: 17 Control: 53	Untargeted	UPLC-ESI-TOF-MS	PCA, hierarchical cluster analysis	37	Low	Low	2	13	—	—	—
Wang L 2013	China	Tissue	Early: 28 Late: 71 Control: 30	Untargeted	1H-NMR	OPLS-DA	35	Low	High	2	7	—	—	—
Jin 2014	China	Plasma	Early: 49 Late: 31 Control: 30	Untargeted	GC-MS	Model of 3 compounds based on OPLS-DA model	33	Low	Low	2	16	90	96.67	0.964
Ma 2014	China	Plasma	Early: 51 Control: 60	Targeted	HPLC	Student *t*-test, PLS-DA	32	Low	Low	2	10	—	—	—
Wang J 2016	China	Plasma	Early: 28 Late: 30 Control: 105	Untargeted	UHPLC-QTOF/MS	Model of 16 compounds based on random forest model	37	Low	Low	1	19	85	90.5	0.929
Xu 2016	China	Urine	Late: 40 Control: 62	Untargeted	LC-MS/MS	Model of 7 compounds based on binary logistic regression and ROC curve	25	Unclear	Low	2	19	90.2	96.0	0.961
Cheng 2017	China	Plasma	Patient: 40 Control: 27	Targeted	LC-MS/MS	Model of 4 compounds based on fivefold cross-validation test	32	Low	Low	2	15	77.5	85.33	0.798
Zhang 2017	China	Plasma	Early: 17 Late: 23 Control: 40	Untargeted	1H-NMR, UHPLC	Model of 9 compounds based on binary logistic regression and ROC curves	33	Low	Low	2	14	97.4	95	0.988

Studies of lung squamous cell carcinoma														
Song 2010	China	Breath	Early: 20 Late: 33 Control: 41	Untargeted	SPME GC-MS	Wilcoxon rank sum test, ROC	29	Low	Low	2	11	—	—	—
De Castro 2014	Spain	Plasma	Patient: 30 Control: 35	Targeted	GC-MS	Model of 1 compound based on ROC curves	30	Unclear	Low	1	13	77	66	0.7
Handa 2014	Germany	Breath	Early: 19 late: 31 Normal: 39	Untargeted	IMS	Model of 11 compounds based on decision tree algorithm	27	Unclear	Low	3	6	97.4	60	—
Rocha 2015	Portugal	Tissue	Patient: 19 Control: 37	Untargeted	1H-NMR	PLS-DA, Wilcoxon rank sum test	25	Unclear	Low	2	7	—	—	—
Sanchez-Rodriguez 2015	Spain	Plasma	Late: 18 Control: 50	Targeted	GC-MS	Model of 1 compound based on ROC curves	31	Low	Low	1	17	69	68	0.68

Studies of head and neck squamous cell carcinoma														
Mizukawa 1998	Japan	Saliva	Patient: 18 Control: 18	Targeted	HPLC	Nil–peak detection only	21	Low	High	1	7	—	—	—
Somashekar 2011	USA	Tissue	Patient: 22 Control: 22	Untargeted	HR-magic angle spinning proton NMR spectroscopy	PCA	23	Low	Low	1	8	—	—	—
Wei 2011	China	Saliva	Early: 21 Late: 16 Control: 66	Untargeted	UPLC-QTOF-MS	Model of 5 compounds based on ROC curves	31	Low	Low	3	13	86.5	82.4	0.89
Yonezawa 2013	Japan	Tissue, plasma	Early: 7 Late: 10 Control: 22	Untargeted	GC-MS	Student's *t*-test, Bartlett's test, Wilcoxon rank sum test	27	Low	Low	2	17	—	—	—
Gruber 2014	Israel	Breath	Early: 9 Late: 11 Control: 40	Untargeted	GC-MS, sensors	Model of 3 compounds based on discriminant factor analysis	30	Low	Low	3	10	77	90	0.83
Wang Q (Clinica Chimica Acta) 2014	China	Saliva	Early: 13 Late: 17 Control: 0	Targeted	UPLC-MS	Model of 4 compounds based on ROC curves	30	Unclear	Low	1	24	92.3	91.7	—
Wang Q (Scientific Reports) 2014	China	Saliva	Early: 13 Late: 17 Control: 30	Untargeted	RPLC-MS, HILIC-MS	Model of 5 compounds based on ROC curve	24	Unclear	Low	1	16	100	96.7	0.997
Wang Q (Talanta) 2014	China	Saliva	Early: 13 Late: 17 Control: 60	Targeted	UPLC-ESI-MS	Model of 2 compounds based on logistic regression model	25	Low	Unclear	1	25	92.3	91.7	0.871
Gupta 2015	India	Plasma	Early: 28 Late: 72 Control: 175	Untargeted	H-NMR	Model of 2 compounds based on OPLS-DA	33	Unclear	Low	2	10	90	94	0.979
Szabo 2015	Hungary	Breath	Cancer: 14 Control: 11	Targeted	OralChroma and GC-MS	Nil–peak detection only	22	Unclear	Low	1	8	—	—	—
Kekatpure 2016	India	Urine	Early: 14 Late: 64 Control: 94	Untargeted	LC-triple quadrupole-MS/MS	Kruskal-Wallis, Fisher exact test, Cox proportional hazards model	23	Low	High	2	13	—	—	—
Mukherjee 2016	USA	Tissue, saliva	Early: 2 Late: 5 Control: 7	Untargeted	LC-MS, LC-MS/MS, GC-MS	Kruskal-Wallis with adjustment for multiple testing	36	Low	Low	3	15	—	—	—
Shoffel-Havakuk 2016	Israel	Saliva	Cancer: 6 Control: 4	Untargeted	GC-MS	Mann–Whitney *U*, Fisher exact test	24	Low	Low	2	11	—	—	—
Bouza 2017	Spain	Breath	Early: 11 Late: 15 Control: 26	Untargeted	SPME, GC-MS	Kruskal-Wallis, Mann–Whitney, PLS-DA, SIMCA prediction	25	Unclear	Low	2	10	—	—	—
Hartwig 2017	Germany	Breath	Early: 5 Late: 5 Control: 4	Untargeted	GC-MS	Jackknife/leave-one-out cross-validation	34	Unclear	Low	3	6	—	—	—
Kamarajan 2017	USA	Tissue, saliva, plasma	Early: 17 Late: 30 Control: 19	Untargeted	UPLC-MS/MS, GC-MS	Anova, *t*-test, random forest classification, PCA	31	Low	Low	2	20	—	—	—
Ohshima 2017	Japan	Saliva	Early: 14 Late: 8 Control: 21	Untargeted	CE-TOF-MS	Hierarchical cluster analysis, Wilcoxon rank sum test	37	Low	Low	3	9	*—*	*—*	—

Key: LC: liquid chromatography; GC: gas chromatography; UPLC: ultra-performance liquid chromatography; HPLC: high-performance liquid chromatography; QTOF: quad-time-of-flight; 1H-NMR: proton nuclear magnetic resonance; UHPLC: ultra-high performance liquid chromatography; IMS: ion mobility spectroscopy; ESI: electrospray ionisation; SPME: solid-phase microextraction; CE: capillary electrophoresis; RPLC: reverse-phase liquid chromatography; HILIC: hydrophilic interaction chromatography; MS: mass spectrometry; PCA: principal component analysis; PLS-DA: partial least squares discriminant analysis; MCCV: Monte Carlo cross-validation; OPLS-DA: orthogonal partial least squares discriminant analysis; ROC: receiver operating curve.

## Supplementary Materials

Supplementary Materials Supplementary Information Supplementary Figure 1: proportion of identified compound classes in different biosamples. Supplementary Table 1: summary of quality assessments. Supplementary Table 2: quality assessment of metabolic metadata based on CAWG-MSI guidelines. Supplementary Table 3: list of all metabolites, chemical class, and studies that identified them to be increased or decreased in biosamples. Supplementary Table 4: summary of significantly different metabolites listed in Supplementary [Supplementary-material supplementary-material-1].
