# Supplementary material for: Metabolic Biomarkers of Squamous Cell Carcinoma of the Aerodigestive Tract: A Systematic Review and Quality Assessment
Source: Oxid Med Cell Longev. 2020 Feb 21;2020:2930347. doi: 10.1155/2020/2930347 (PMC7330643; doi:10.1155/2020/2930347)
Supplement: Supplementary Materials — Supplementary Information Supplementary Figure 1: proportion of identified compound classes in different biosamples. Supplementary Table 1: summary of quality assessments. Supplementary Table 2: quality assessment of metabolic metadata based on CAWG-MSI guidelines. Supplementary Table 3: list of all metabolites, chemical class, and studies that identified them to be increased or decreased in biosamples. Supplementary Table 4: summary of significantly different metabolites listed in Supplementary . [file 2930347.f1.docx]

## Supplementary Information

Supplementary Figure 1: Proportion of identified compound classes in different biosamples 2

Supplementary Table 1: Summary of quality assessments 5

Supplementary Table 2: Quality assessment of metabolic metadata based on CAWG-MSI guidelines. 9

Supplementary Table 3: List of all metabolites, chemical class, studies that identified them to be increased or decreased in biosamples. 21

Supplementary Table 4: Summary of significantly different metabolites list in Supplementary Table 3 23

Supplementary Figure 1: Proportion of identified compound classes in different biosamples

|  | **Author** | **Overall diagnostic Quality** | **STARD score** | **QUADAS** | | | | | | | | **CAWG-MSI metadata** |
| --- | --- | --- | --- | --- | --- | --- | --- | --- | --- | --- | --- | --- |
|  |  |  |  | **Risk of bias** | | | | | **Applicability concerns** | | |  |
|  |  |  |  | **Patient selection** | **Index test** | **Reference standard** | **Flow and timing** | **Patient selection** | | **Index** | **Reference standard** |  |
| OSCC | Cheng 2017 | Good | 32 | Low | Low | Low | Low | Low | | Low | Low | 15 |
|  | Jin 2014 | Good | 33 | Low | Low | Low | Low | Low | | High | Low | 16 |
|  | Liu 2013 | Good | 37 | Low | Low | Low | Low | Low | | Low | Low | 13 |
|  | Ma 2014 | Fair | 32 | Low | Low | Low | Low | Low | | Low | Low | 10 |
|  | Wang J 2016 | Good | 37 | Low | Low | Low | Low | High | | low | Low | 19 |
|  | Wang L 2013 | Good | 35 | Low | Low | Low | Low | High | | High | Low | 7 |
|  | Xu 2016 | Fair | 25 | High | Low | Low | Unclear | Low | | Low | Low | 19 |
|  | Zhang 2017 | Good | 33 | Low | Low | Low | Unclear | Low | | Low | Low | 14 |
| LSCC | De Castro 2014 | Fair | 30 | Low | Low | Unclear | Unclear | Low | | Low | Low | 13 |
|  | Handa 2014 | Fair | 27 | High | Low | Unclear | Unclear | Low | | Low | High | 6 |
|  | Rocha 2015 | Fair | 25 | Low | Unclear | Low | Unclear | Low | | Low | Low | 7 |
|  | Sanchez-Rodriguez 2015 | Fair | 31 | Low | Low | Low | Unclear | Low | | Low | Low | 17 |
|  | Song 2010 | Fair | 29 | Low | Low | Low | Low | Unclear | | Low | Low | 11 |
| HNSCC | Gupta 2015 | Good | 33 | Unclear | Low | Low | High | Low | | low | Low | 10 |
|  | Hartwig 2017 | Good | 34 | Low | High | Unclear | Low | Low | | Low | Low | 6 |
|  | Kekatpure 2016 | Poor | 23 | Unclear | Low | Low | Low | High | | High | High | 13 |
|  | Mizukawa 1998 | Poor | 21 | High | Low | Low | Low | Low | | High | High | 7 |
|  | Ohshima 2017 | Good | 37 | Low | Low | Low | Low | Low | | Low | Low | 9 |
|  | Szabo 2015 | Poor | 22 | Unclear | Unclear | Low | Unclear | Low | | Low | High | 8 |
|  | Wang Q (Talanta) 2014 | Fair | 25 | Unclear | Low | Low | Low | Unclear | | Low | Unclear | **25** |
|  | Wang Q (Scientific reports) 2014 | Poor | 24 | High | Low | Low | Unclear | Low | | Low | Low | 16 |
|  | Wang Q (Clinica Chimica Acta) 2014 | Fair | 30 | High | Low | Low | Unclear | Low | | Low | Low | **24** |
|  | Wei 2011 | Fair | 31 | Low | Low | Low | Low | Low | | Low | Low | 13 |
|  | Bouza 2017 | Fair | 25 | Unclear | Low | Low | Unclear | Low | | Low | Low | 10 |
|  | Kamarajan 2017 | Fair | 31 | Low | Low | Low | Low | Low | | Low | Low | 20 |
|  | Mukherjee 2017 | Good | 36 | Low | Low | Low | Low | Low | | Low | Low | 15 |
|  | Shoffel-Havakuk 2016 | Poor | 24 | Low | Low | Low | Low | Low | | Low | Low | 11 |
|  | Somashekar 2011 | Poor | 23 | Low | Low | Low | Unclear | Low | | Low | Low | 8 |
|  | Yonezawa 2013 | Fair | 27 | Low | Low | Low | Low | Low | | Low | Low | 17 |
|  | Gruber 2014 | Fair | 30 | Unclear | Low | Low | Low | Low | | Low | Low | 10 |

Supplementary Table 1: Summary of quality assessments

|  | Sample preparation | | | | | | Experimental analysis | | Instrumental performance | | | | Method validation | | | | | Metabolite identification | | | | | | | | | | Data pre-processing | | Metabolite identification |
| --- | --- | --- | --- | --- | --- | --- | --- | --- | --- | --- | --- | --- | --- | --- | --- | --- | --- | --- | --- | --- | --- | --- | --- | --- | --- | --- | --- | --- | --- | --- |
| Author | Biological replicates | Tissue Harvesting | Biofluid harvesting | Tissue processing | Storage condition | Extraction method | Instrument description | Separation column | Instrument description | Sample introduction & delivery | Ionisation source | Mass analyser description and acquisition mode | m/z calibration standard | QC samples | Internal Standards used | Retention time markers | Accuracy and precision | Relative quantification | | | Absolute quantification | | | | | | | Post acquisition data pre-processing | |  |
|  |  |  |  |  |  |  |  |  |  |  |  |  |  |  |  |  |  | Description and quantifer of IS metabolite | Description of method used for asessing instrument response | Replicate analyses | Calibration curves for each metabolite | range of standards used | Quantification of method accuracy | Quantification of method precision | LLOQ | LLOD | Recovery/ stability | Data file format/ conversion methods | Data-preprocessing methods | Level |
| Wang J 2016 | N |  | Y |  | NA | Y | Y | Y | Y | Y | Y | Y | N | Y | N | N | NA | NA | N | Y |  |  |  |  |  |  |  | Y | Y | 1 |
| Wang L 2013 | N | Y |  | Y | Y | Y | Y | NA | NA | NA | NA | NA | N - NMR instrument performance verification parameters not met | | | |  | N | N | N |  |  |  |  |  |  |  | N | N | 2 |
| Xu 2016 | N |  | Y |  | Y | NA | Y | Y | Y | Y | Y | Y | Y | Y | Y | Y | N | Y | Y | N |  |  |  |  |  |  |  | Y | N | 2 |
| Cheng 2017 | N |  | Y |  | Y | Y | Y | Y | Y | Y | Y | Y | N | N | Y | N | N | Y | Y | N |  |  |  |  |  |  |  | N | N | 2 |
| Jin 2014 | N |  | Y |  | Y | Y | Y | Y | Y | Y | Y | Y | N | Y | Y | N | N | N | Y | N |  |  |  |  |  |  |  | N | N | 2 |
| Liu 2013 | N |  | Y |  | Y | Y | Y | Y | Y | Y | Y | Y | N | N | N | N | N | N | Y | N |  |  |  |  |  |  |  | N | Y | 2 |
| Ma 2014 | N |  | Y |  | Y | Y | Y | Y | Y | N | N | N | Y | NA | NA | Y | N |  |  |  | Y | N | N | N | N | N | N | N | N | 2 |
| Zhang 2017 | N |  | Y |  | Y | Y | Y | Y | Y | Y | Y | N | N | NA | NA | N | N |  |  |  | Y | Y | N | N | N | N | N | N | N | 2 |
| Rocha 2015 | N | Y |  | Y | Y | N | Y | NA | NA | NA | NA | NA | N – NMR instrument performance verification parameters not met | | | |  | N | Y | N |  |  |  |  |  |  |  | N | Y | 2 |
| Sanchez-Rodriguez 2015 | Y |  | Y |  | Y | Y | Y | Y | Y | Y | Y | N | N | N | N | N | Y |  |  |  | Y | Y | N | N | N | N | N | N | N | 1 |
| Song 2010 | Y |  | NA |  | N | NA | Y | N | Y | Y | Y | N | N | Y | N | N | N |  |  |  | Y | Y | N | N | N | N | N | N | N | 2 |
| De Castro 2014 | N |  | Y |  | Y | Y | Y | Y | Y | N | Y | N | N | N | N | N | N |  |  |  | Y | Y | Y | Y | N | N | N | N | N | 1 |
| Handa 2014 | N |  | NA |  | N | NA | Y | Y | Y | Y | Y | N | N | N | N | N | N | N | N | N |  |  |  |  |  |  |  | N | N | 3 |
| Szabo 2015 | Y |  | NA |  | N | NA | Y | N | Y | Y | N | N | N | N | N | N | N |  |  |  | Y | Y | Y | Y | N | N | N | N | N | 1 |
| Wang Q (Talanta) 2014 | N |  | Y |  | Y | Y | Y | Y | Y | N | Y | Y | Y | N | N | Y | Y |  |  |  | Y | Y | Y | Y | Y | Y | Y | N | N | 1 |
| Wang Q (Scientific reports) 2014 | N |  | Y |  | Y | Y | Y | Y | Y | Y | Y | Y | N | N | N | N | N | N | Y | N |  |  |  |  |  |  |  | N | Y | 1 |
| Wang Q (Clinica Chimica Acta) 2014 | N |  | Y |  | Y | Y | Y | Y | Y | Y | Y | Y | N | N | N | Y | N |  |  |  | Y | Y | Y | Y | Y | Y | Y | N | N | 1 |
| Wei 2011 | N |  | Y |  | Y | N | Y | Y | Y | N | Y | Y | N | N | N | N | N | N | Y | N |  |  |  |  |  |  |  | N | Y | 3 |
| Bouza 2017 | N |  | NA |  | Y | NA | Y | Y | Y | N | Y | N | N | N | N | N | N | N | Y | N |  |  |  |  |  |  |  | N | Y | 2 |
| Gupta 2015 | N |  | Y |  | Y | N | Y | Y | Y | Y | Data acquisition parameters - Y | | Y - NMR instrument parameters met | | |  |  | N | N | N |  |  |  |  |  |  |  | N | Y | 2 |
| Hartwig 2017 | N |  | NA |  | N | NA | Y | Y | Y | Y | N | N | N | N | N | N | N | N | N | N |  |  |  |  |  |  |  | N | N | 3 |
| Kekatpure 2016 | N |  | Y |  | Y | Y | Y | Y | Y | Y | Y | Y | N | N | N | N | N |  |  |  | N | N | N | N | N | N | N | N | N | 2 |
| Mizukawa 1998 | N |  | N |  | Y | N | Y | Y | Y | N | N | N | N | N | N | N | N |  |  |  | Y | Y | N | N | N | N | Y | N | N | 1 |
| Ohshima 2017 | N |  | Y |  | Y | Y | Y | Y | Y | N | Y | N | N | N | N | N | N | N | N | N |  |  |  |  |  |  |  | N | Y | 3 |
| Shoffel-Havakuk 2016 | N |  | N |  | N | Y | Y | Y | Y | Y | N | Y | N | N | N | N | N | N | N | N |  |  |  |  |  |  |  | N | Y | 2 |
| Somashekar 2011 | N | Y |  | Y | Y | N | Y | NA | NA | NA | NA | NA | N – NMR instrument performance verification parameters not met | | | |  | Y | N | N |  |  |  |  |  |  |  | N | Y | 1 |
| Yonezawa 2013 | N | Y | Y | Y | Y | Y | Y | Y | Y | N | N | Y | N | N | Y | N | N | Y | Y | N |  |  |  |  |  |  |  | Y | Y | 2 |
| Gruber 2014 | Y |  | NA |  | Y | Y | Y | Y | Y | Y | N | N | N | N | N | N | N | N | N | N |  |  |  |  |  |  |  | N | N | 3 |
| Kamarajan 2017 | Y | Y | Y | Y | Y | N | Y | Y | Y | Y | Y | Y | N | Y | N | N | Y | N | Y | N |  |  |  |  |  |  |  | N | Y | 2 |
| Mukherjee 2017 | N | Y | N | Y | Y | Y | Y | Y | Y | N | Y | Y | Y | N | N | N | N | N | Y | N |  |  |  |  |  |  |  | N | N | 3 |

Supplementary Table 2: Quality assessment of metabolic metadata based on CAWG-MSI guidelines. Key: Y: yes, N: no, NA: not applicable. LLOQ: lower limits of quantification, LLOD: lower limits of detection, NMR: nuclear magnetic resonance

| **Metabolites identified** |  |  | **Tissue** | | **Plasma** | **Urine** | **Saliva** | | **Breath** |
| --- | --- | --- | --- | --- | --- | --- | --- | --- | --- |
| **Compound name** | **Chemical Classes** | **Studies** | **Increased/decreased in SCC** | | | | | | |
| Ethanol | alcohols/ phenols | Wang L 2013 | Decreased | |  |  | |  | Increased |
| 2-aminoethanol |  | Yonezawa 2013 | Increased | |  |  | |  |  |
| Glycerol |  | Yonezawa 2013 | Increased | |  |  | |  |  |
| 1-butanol |  | Song 2010 |  | |  |  | |  | Increased |
| Coniferyl aldehyde | aldehyde | Yonezawa 2013 | Decreased | |  |  | |  |  |
| Undecane | alkane | Bouza 2017 |  | |  |  | |  | Increased |
| 1-Heptene | alkene | Hartwig 2017 |  | |  |  | |  | Increased |
| L-aspartate | Amino acid | Wang L 2013 | Decreased | |  |  | |  |  |
| Creatinine |  | Wang L 2013, Jin 2014 | Decreased | | Decreased |  | |  |  |
| Glutamine |  | Wang L 2013, Zhang 2017, Somashekar 2011, Jin 2014 | Decreased | | Decreased |  | |  |  |
| Benzylmetcapturic acid |  | Yonezawa 2013 | Decreased | |  |  | |  |  |
| Beta-N-methyl-amino-L-alanine |  | Yonezawa 2013 | Decreased | |  |  | |  |  |
| Cysteine |  | Jin 2014, Ma 2014, Yonezawa 2013 | Increased | | Decreased |  | |  |  |
| Tryptophan |  | Jin 2014, Ohshima 2017 | Increased | | Decreased |  | | Increased |  |
| Alanine |  | Rocha 2015, Somashekar 2011, Yonezawa 2013, Mukherjee 2016, Jin 2014, Ma 2014, Ohshima 2017, Wei 2011 | Increased | | Decreased |  | | Increased |  |
| Aspartate |  | Somashekar 2011, Ma 2014 | Increased | | Decreased |  | |  |  |
| Tyrosine |  | Somashekar 2011, Yonezawa 2013, Chang 2017, Klupczynska 2017 | Increased | | Decreased |  | |  |  |
| Histidine |  | Somashekar 2011, Yonezawa 2013, Klupczynska 2017, Ma 2014 | Increased | | Decreased |  | |  |  |
| Lysine |  | Somashekar 2011, Yonezawa 2013, Ma 2014 | Increased | | Decreased |  | | Increased |  |
| Glycine |  | Wang L 2013, Somashekar 2011, Yonezawa 2013, Mukherjee 2016, Ma 2014 | Increased | | Decreased |  | |  |  |
| L-Tyrosine |  | Wang L 2013, Wang J 2016 | Increased | | Decreased |  | |  |  |
| Methionine |  | Wang L 2013, Yonezawa 2013, Mukherjee 2016, Ma 2014, Klupczynska 2017 | Increased | | Decreased |  | |  |  |
| Glutamate |  | Wang L 2013, Zhang 2017, Rocha 2015, Somashekar 2011, Ma 2014 | Increased | | Decreased |  | |  |  |
| Phenylalanine |  | Wang L 2013, Zhang 2017, Somashekar 2011, Yonezawa 2013, Chang 2017, Ma 2014, Wei 2011, Wang Q Talenta | Increased | | Decreased |  | | Decreased |  |
| Citruline |  | Yonezawa 2013 | Increased | |  |  | |  |  |
| Homoserine |  | Yonezawa 2013 | Increased | |  |  | |  |  |
| Sarcosine |  | Yonezawa 2013 | Increased | |  |  | |  |  |
| Asparagine |  | Yonezawa 2013, Ma 2014 | Increased | | Decreased |  | |  |  |
| Threonine |  | Yonezawa 2013, Mukherjee 2016, Jin 2014, Ma 2014, Wei 2011 | Increased | | Decreased |  | |  |  |
| Ornithine |  | Yonezawa 2013, Wang Q Scientific reports | Increased | |  |  | | Increased |  |
| Kynurenine |  | Zhang 2017 | Increased | |  |  | |  |  |
| Acetylcarnitine |  | Zhang 2017, Mukherjee 2016, Xu 2016 | Increased | |  | Increased | |  |  |
| Pyroglutamic acid |  | Zhang 2017, Yonezawa 2013, Klupczynska 2017, Xu 2016 | Increased | |  | Increased | |  |  |
| Proline |  | Zhang 2017, Yonezawa 2013, Ma 2014, Wei 2011 | Increased | |  |  | |  |  |
| Taurine |  | Wang L 2013, Zhang 2017, Somashekar 2011, Yonezawa 2013, Mukherjee 2016, Ma 2014, Ohshima 2017 | Increased* | | Decreased |  | | Increased |  |
| Hydroxyproline |  | Yonezawa 2013 | Increased* | |  |  | |  |  |
| Levodopa |  | Chang 2017 |  | | Increased |  | |  |  |
| Beta-alanine |  | Jin 2014 |  | | Increased |  | |  | Increased |
| Arginine |  | Ma 2014 |  | | Decreased |  | |  |  |
| HNP-1 |  | Mizukawa 1998 |  | |  |  | | Increased |  |
| Trimethyllysine |  | Ohshima 2017 |  | |  |  | | Increased |  |
| Tryptophan |  | Wang J 2016 |  | | Decreased |  | |  |  |
| Phenylacetylglutamine |  | Wang L 2013, Mukherjee 2016, Xu 2016, Klupczynska 2017 |  | |  | Increased | |  |  |
| Carbocysteine |  | Wang Q Scientific reports |  | |  |  | | Decreased |  |
| Acetylphenylalanine |  | Wang Q Scientific reports, Mukherjee 2016 |  | |  |  | |  | Decreased |
| Urocanic acid |  | Xu 2016 |  | |  | Increased | |  |  |
| Serine |  | Zhang 2017, Yonezawa 2013, Ma 2014 |  | |  |  | | Increased |  |
| Isoleucine | BCAA | Wang L 2013, Somashekar 2011, Ma 2014, Ohshima 2017, Wei 2011 | Increased | | Decreased | Increased | |  |  |
| Valine |  | Wang L 2013, Zhang 2017, Somashekar 2011, Yonezawa 2013, Mukherjee 2016, Jin 2014, Ma 2014, Ohshima 2017, Wei 2011 | Increased | | Decreased |  | | Increased |  |
| Leucine |  | Wang Q Talenta, Wang L 2013, Somashekar 2011, Ma 2014, Klupczynska 2017, Ohshima 2017, Wei 2011 | Increased | | Decreased |  | | Increased |  |
| Trehalose | Carbohydrate | Yonezawa 2013 | Increased | |  |  | |  |  |
| Maltose |  | Jin 2014 |  | | Increased |  | |  |  |
| Melibiose |  | Yonezawa 2013 |  | | Decreased |  | |  |  |
| Glucose |  | Wang L 2013, Rocha 2015, Yonezawa 2013, Jin 2014 | Decreased | | Decreased |  | |  |  |
| Fructose |  | Mukherjee 2016 |  | |  | Increased | |  |  |
| L-Fucose |  | Xu 2016 |  | |  | Increased | |  |  |
| Glycoprotein |  | Wang L 2013 | Increased | |  |  | |  |  |
| Butyric acid | Carboxylic acid | Schoffel 2016, Ohshima 2017 | Increased | | Increased |  | | Increased |  |
| Hippurate |  | Wang L 2013 | Increased | |  |  | |  |  |
| Gamma-aminobutyric acid (GABA) |  | Wang L 2013, Mukherjee 2016, Jin 2014, Wei 2011 | Increased | |  |  | | Decreased |  |
| Acetoacetic acid |  | Wang L 2013, Yonezawa 2013 | Increased | |  |  | |  |  |
| Malonate |  | Wang L 2013, Yonezawa 2013 | Increased | |  |  | |  |  |
| 2-aminobutyric acid |  | Yonezawa 2013 | Increased | |  |  | |  |  |
| 2-dehydro-D-gluconate |  | Yonezawa 2013 | Increased | |  |  | |  |  |
| 3,4-dihydroxyphenylacetic acid (DOPAC) |  | Chang 2017 |  | | Increased |  | |  |  |
| Propionate |  | Gupta 2015 |  | | Increased |  | |  |  |
| alpha-hydroxybutyric acid |  | Jin 2014 |  | |  |  | | Increased |  |
| alpha-hydroxyisocaproic acid |  | Ohshima 2017 |  | |  |  | | Increased |  |
| Fumaric acid |  | Jin 2014 |  | | Decreased |  | | Increased |  |
| Iminodiacetic acid |  | Jin 2014 |  | | Increased |  | |  |  |
| Indolelactic acid |  | Jin 2014 |  | | Decreased |  | |  |  |
| beta-hydroxybutyric acid |  | Jin 2014, Ohshima 2017 |  | |  |  | | Increased |  |
| PGE-M |  | Kekatpure 2016 |  | |  | Increased | |  |  |
| 4-hydroxyphenylacetic acid |  | Ohshima 2017 |  | |  |  | | Increased |  |
| Phenyllactic acid |  | Ohshima 2017 |  | |  |  | | Increased |  |
| Phenylpropionic acid |  | Ohshima 2017 |  | |  |  | | Increased |  |
| Phloretic acid |  | Ohshima 2017 |  | |  |  | | Increased |  |
| Pentanoic acid |  | Schoffel 2016 |  | |  |  | | Increased |  |
| Heptanoic acid |  | Schoffel 2016, Ohshima 2017 |  | |  |  | | Increased |  |
| Hexanoic acid |  | Schoffel 2016, Ohshima 2017 |  | |  |  | | Increased |  |
| Gamma-hydroxyglutamic acid |  | Wang Q Scientific reports |  | |  |  | | Decreased |  |
| 3-hydroxyisovaleric acid |  | Yonezawa 2013 |  | |  | Decreased | |  |  |
| Pipecolic acid |  | Yonezawa 2013, Wang Q Clinica Chimica Acta |  | | Decreased |  | |  |  |
| Formate | Ester | Wang L 2013 | Increased | |  |  | |  |  |
| Acetate |  | Wang L 2013, Rocha 2015, Somashekar 2011, Gupta 2015 | Increased | | Increased |  | |  |  |
| Undecanoylcarnitine (11:1) |  | Xu 2016 |  | |  | Increased | |  |  |
| Stearic acid | Fatty Acid | De Castro 2014 | Increased | |  |  | |  |  |
| Oleic Acid |  | Jin 2014, Wang J 2016 | Increased | | Increased |  | |  |  |
| Arachidonic acid |  | Mukherjee 2016, Zhang 2017, Sanchez-Rodriguez 2015 | Increased | | Decreased |  | |  |  |
| Docosapentaenoic acid |  | Sanchez-Rodriguez 2015 | Increased | |  |  | |  |  |
| Margaric acid |  | Sanchez-Rodriguez 2015 | Increased | |  |  | |  |  |
| Eicosenoic acid |  | Sanchez-Rodriguez 2015, Wei 2011 | Increased | | Decreased |  | |  |  |
| Oleic acid |  | De Castro 2014, Sanchez-Rodriguez 2015, Mukherjee 2016 |  | | Decreased |  | |  |  |
| Hexadecanoic acid/palmitic acid |  | Jin 2014, Wang J 2016 |  | | Increased |  | |  |  |
| Octanoic acid |  | Ohshima 2017 |  | |  |  | | Increased |  |
| Arachidic acid |  | Sanchez-Rodriguez 2015 |  | | Decreased |  | |  |  |
| Docosanoic acid |  | Sanchez-Rodriguez 2015 |  | | Decreased |  | |  |  |
| Heneicosanoic acid |  | Sanchez-Rodriguez 2015 |  | | Decreased |  | |  |  |
| Heptadecaenoic acid |  | Sanchez-Rodriguez 2015 |  | | Decreased |  | |  |  |
| Linoleic acid |  | Sanchez-Rodriguez 2015 |  | | Decreased |  | |  |  |
| Palmitic acid |  | Sanchez-Rodriguez 2015 |  | | Decreased |  | |  |  |
| Pentadecanoic acid |  | Sanchez-Rodriguez 2015 |  | | Decreased |  | |  |  |
| Myristic acid |  | Sanchez-Rodriguez 2015, Jin 2014, Wei 2011 |  | | Decreased |  | |  |  |
| Eicosapentaenoic acid |  | Sanchez-Rodriguez 2015, Mukherjee 2016 |  | | Decreased |  | |  |  |
| Palmitoleic acid |  | Sanchez-Rodriguez 2015, Mukherjee 2016 |  | | Decreased |  | |  |  |
| Palmitoleic acid |  | Wang J 2016 |  | | Increased |  | |  |  |
| Dodecanoic acid |  | Wang J 2016, Wei 2011 |  | | Decreased |  | |  |  |
| Nonanoic acid |  | Xu 2016, Mukherjee 2016 |  | |  | Decreased | |  |  |
| Octenoylcarnitine |  | Xu 2016, Mukherjee 2016 |  | |  | Decreased | |  |  |
| 2-hydroxyvaleric acid |  | Ohshima 2017 |  | |  |  | | Increased |  |
| Linoleic acid |  | Jin 2014, Wang J 2016, Mukherjee 2016 |  | | Increased |  | |  |  |
| Phenylacetylglycine | Hydrocarbon | Wang L 2013 | Decreased | |  |  | |  |  |
| 2-hydroxypyridine |  | Yonezawa 2013 | Decreased | |  |  | |  |  |
| Alpha-sorbopyranose |  | Yonezawa 2013 | Decreased | |  |  | |  |  |
| 1,3-propanediamine |  | Yonezawa 2013 | Increased | |  |  | |  |  |
| Xylene |  | Bouza 2017 |  | |  |  | |  | Increased |
| Dibutylhydroxytoluene |  | Hartwig 2017 |  | |  |  | |  | Increased |
| P-xylene |  | Hartwig 2017 |  | |  |  | |  | Decreased |
| Toluene |  | Hartwig 2017, Bouza 2017 |  | |  |  | |  | Increased |
| Terephthalic acid |  | Ohshima 2017 |  | |  |  | | Increased |  |
| Cortisol |  | Wang J 2016 |  | | Increased |  | |  |  |
| Decanoylcarnitine (10:0) |  | Xu 2016 |  | |  | Decreased | |  |  |
| Phosphate | Inorganic compound | Yonezawa 2013 | Increased | |  |  | |  |  |
| Phosphoric acid |  | Zhang 2017 |  | | Decreased |  | |  |  |
| alpha-ketoisocaproic acid (KIC) | Ketoacid | Jin 2014, Mukherjee 2016 |  | | Decreased |  | |  |  |
| alpha-ketoisovaleric acid (KIV) |  | Jin 2014, Ohshima 2017 |  | | Decreased |  | | Increased |  |
| alpha-ketomethylvaleric acid (KMV) |  | Jin 2014 |  | | Decreased |  | |  |  |
| Acetone | Ketone | Wang L 2013, Gupta 2015 | Increased | | Increased |  | |  |  |
| 3-hydroxy-2-butanone |  | Song 2010 |  | |  |  | |  | Increased |
| Phosphoethanolamine | Lipid | Rocha 2015, Jin 2014 | Increased | | Decreased |  | |  |  |
| Phosphocholine |  | Rocha 2015, Somashekar 2011 | Increased | |  |  | |  |  |
| O-phosphoethanolamine |  | Yonezawa 2013 | Increased | | Decreased |  | |  |  |
| LysoPC (15:0) |  | Zhang 2017 | Increased | |  |  | |  |  |
| Cholesterol |  | Jin 2014 |  | | Decreased |  | |  |  |
| LPA (18:1/0:0) |  | Wang J 2016 |  | | Decreased |  | |  |  |
| LysoPC (14:0) |  | Wang J 2016 |  | | Decreased |  | |  |  |
| LysoPC (18:4) |  | Wang J 2016 |  | | Decreased |  | |  |  |
| LysoPC (24:0) |  | Wang J 2016 |  | | Increased |  | |  |  |
| phytosphingosine |  | Wang Q Scientific reports |  | |  |  | | Decreased |  |
| LysoPC (18:2) |  | Zhang 2017, Wang J 2016 |  | | Increased |  | |  |  |
| Adenosine | Nitrogen compound | Mukherjee 2016 | Increased | |  |  | |  |  |
| Acetyl hydrazine |  | Wang L 2013 | Increased | |  |  | |  |  |
| Trimethylamine |  | Wang L 2013 | Increased | |  |  | |  |  |
| Putrescine |  | Yonezawa 2013 | Increased | |  |  | |  |  |
| Carnitine |  | Klupczynska 2017, Wang Q Scientific Reports, Xu 2016 |  |  | | Increased | | Decreased |  |
| Cadaverine |  | Ohshima 2017 |  | |  |  | | Increased |  |
| Gamma-butyrobetaine |  | Ohshima 2017 |  | |  |  | | Increased |  |
| Urea |  | Ohshima 2017 |  | |  |  | | Decreased |  |
| Betaine |  | Wang Q Clinica Chimica Acta |  | |  |  | | Increased |  |
| Hexanoylcarnitine |  | Wang Q Scientific reports |  | |  |  | | Increased | Increased |
| Heptanoylcarnitine |  | Xu 2016 |  | |  | Decreased | |  |  |
| Indoxyl |  | Xu 2016 |  | |  | Increased | |  |  |
| Adenosine phosphate monosodium (AMP) | Nucleotide | Wang L 2013 | Decreased | |  |  | |  |  |
| Nicotinamide adenine dinucleotide |  | Wang L 2013 | Decreased | |  |  | |  |  |
| Inosine |  | Zhang 2017, Yonezawa 2013 | Decreased | |  |  | |  |  |
| Hypoxanthine |  | Yonezawa 2013 | Increased | |  |  | |  |  |
| Guanosine-3-monophosphate-3 |  | Mukherjee 2016 |  | |  | Increased | |  |  |
| Cyclic guanosine monophosphate (cGMP) |  | Xu 2016 |  | |  | Increased | |  |  |
| Glycolic acid | Organic acid | Yonezawa 2013, Jin 2014, Ohshima 2017 | Decreased | |  |  | | Increased |  |
| Alpha-ketoglutaric acid oxime |  | Wang L 2013 | Decreased | |  |  | |  |  |
| Creatine |  | Wang L 2013, Rocha 2015, Somashekar 2011 | Decreased | |  |  | | Increased |  |
| Lactate |  | Rocha 2015, Somashekar 2011 | Increased | |  |  | | Increased |  |
| Glucarate |  | Yonezawa 2013 | Increased | |  |  | |  |  |
| Lactic acid |  | Jin 2014, Wang Q Scientific reports, Wei 2011 |  | | Increased |  | | Increased |  |
| Hydroxyphenyllactic acid |  | Wang Q Scientific Reports, Chang 2017 |  | | Increased |  | |  |  |
| Myo-inositol | Polyol | Wang L 2013, Jin 2014 | Decreased | |  |  | |  |  |
| Uric acid | Purine | Jin 2014, Xu 2016 |  | | Decreased | Increased | |  |  |
| 2-deoxyuridine | Pyrimidine | Yonezawa 2013 | Decreased | |  |  | |  |  |
| Adenine |  | Wang L 2013, Mukherjee 2016 | Increased | |  |  | |  |  |
| Uracil |  | Wang L 2013, Zhang 2017, Yonezawa 2013 | Increased | |  |  | |  |  |
| Cytosine |  | Yonezawa 2013 | Increased | |  |  | |  |  |
| 5-Methylcytidine |  | Zhang 2017 | Increased | |  |  | |  |  |
| 5-hydroxymethyluracil |  | Wang Q Scientific reports |  | |  |  | | Increased |  |
| Deoxycytidine |  | Xu 2016 |  | |  | Increased | |  |  |
| Decamethylcyclopentasiloxane | Silicon compound | Hartwig 2017 |  | |  |  | |  | Decreased |
| Sulfonic acid | Sulphur compound | Yonezawa 2013 | Increased | |  |  | |  |  |
| Dimethyl disulfide |  | Hartwig 2017, Szabo 2015, Bouza 2017 |  | |  |  | |  | Decreased |
| Methylcysteine |  | Jin 2014 |  | | Decreased |  | |  |  |
| Ascorbic acid | Vitamin | Yonezawa 2013 | Decreased | |  |  | |  |  |
| Nicotinamide |  | Yonezawa 2013 | Increased | |  |  | |  |  |
| Choline |  | Zhang 2017, Gupta 2015, Ohshima 2017, Wang Q Clinica Chimica Acta | Increased | | Increased |  | | Increased |  |

Supplementary Table 3: List of all metabolites, chemical class, studies that identified them to be increased or decreased in biosamples. Key: BCAA: branched chain amino acid

| **Compound name** | **Chemical Classes** | **Metabolic pathway** | **Tisssue** | **Saliva** | **Plasma** | **Urine** | **Breath** |
| --- | --- | --- | --- | --- | --- | --- | --- |
| Alanine | Amino acid | Amino acid metabolism | Decreased |  | Decreased |  |  |
| Creatinine |  |  | Decreased |  | Decreased |  |  |
| Cysteine |  |  | Increased |  | Decreased |  |  |
| Glutamate |  |  | Increased | Increased | Decreased |  |  |
| Glutamine |  |  | Increased | Increased | Decreased |  |  |
| Glycine |  |  | Increased |  | Decreased |  |  |
| Methionine |  |  | Increased |  | Decreased |  |  |
| Ornithine |  |  | Increased |  | Decreased |  |  |
| Proline |  |  | Increased | Increased | Decreased |  |  |
| Taurine |  |  | Increased |  | Decreased | Increased |  |
| Tryptophan |  |  | Increased |  | Decreased |  |  |
| Tyrosine |  |  | Increased |  | Decreased |  |  |
| Acetate | Ester |  | Increased |  | Decreased |  |  |
| Creatine | Organic acid |  | Increased |  | Decreased |  |  |
| Choline | Vitamin |  | Increased | Decreased | Decreased |  |  |
| Asparagine | Amino acid | Amino acid metabolism/Lipid metabolism | Increased | Increased | Decreased |  |  |
| Aspartate |  |  | Increased | Increased | Decreased |  |  |
| Serine |  |  | Increased |  | Decreased |  |  |
| Threonine |  |  | Increased |  | Decreased |  |  |
| Isoleucine | BCAA | BCAA metabolism | Increased | Increased |  |  |  |
| Leucine |  |  | Increased |  |  | Increased |  |
| Valine |  |  | Increased |  |  | Increased |  |
| Acetoacetic acid | Carboxylic acid |  | Increased |  |  |  |  |
| Dodecanoic acid | Fatty acid | Lipid metabolism | Increased | Increased | Decreased |  |  |
| Myristic acid |  |  |  |  |  | Increased |  |
| Palmitoleic acid |  |  |  |  |  |  | Decreased |
| Uracil | Pyrimidine |  |  | Increased |  |  |  |

Supplementary Table 4: Summary of significantly different metabolites list in Supplementary Table 3
